# Impact of scalpel type on operative time and acute complications in thyroidectomies

**DOI:** 10.1016/j.bjorl.2019.08.004

**Published:** 2019-10-03

**Authors:** Tamires Santos Fraga, Hugo Fontan Köhler, Thiago Celestino Chulam, Luiz Paulo Kowalski

**Affiliations:** A.C. Camargo Câncer Center, Departamento de Cirurgia de Cabeça e Pescoço e Otorrinolaringologia, São Paulo, SP, Brazil

**Keywords:** Thyroid gland, Thyroidectomy, Surgical hemostasis, Postoperative complications

## Abstract

**Introduction:**

Thyroidectomy is the most common surgery in the cervical region. Currently, several techniques are available for intraoperative hemostasis.

**Objective:**

To compare the performance of three techniques (monopolar and bipolar electrical and ultrasonic) on operative time and postoperative complications.

**Methods:**

Patients submitted to total thyroidectomy without prior treatment were included in this prospective series study, using a scientific design.

**Results:**

A total of 834 patients were included; 661 women (79.3%) and 173 men (20.7%). The diagnosis was malignant neoplasia in 528 patients (63.3%) and benign disease in 306 patients (36.7%). The monopolar electric scalpel was used in 280 patients (33.6%), bipolar scalpel in 210 patients (25.2%) and ultrasonic scalpel in 344 patients (41.3%). The operative time was significantly shorter with the ultrasonic or bipolar scalpel when compared to the electric scalpel. In a linear regression model, gender, malignancy diagnosis and power energy type were significant for the procedure duration. Patients who underwent surgery with an ultrasound or bipolar scalpel had a significantly lower incidence of hypoparathyroidism.

**Conclusion:**

The use of ultrasonic or bipolar scalpel significantly reduces operative time and the incidence of transient hypoparathyroidism.

## Introduction

The use of advanced ultrasound-based bipolar devices for sealing vessels in surgical procedures can result in a significant reduction in operative time.^1^ The reduction in operative time, besides being desirable because it allows better use of hospital resources such as the operating room itself, also has a significant impact on the reduction of postoperative complications.^2^

Thyroidectomy is the most frequently performed procedure in head and neck surgeries. As in any surgical procedure, this surgery has risks and complications, with intra- or postoperative hemorrhage being its main acute adverse effect.^3^ Multiple techniques can be used to perform intraoperative hemostasis, such as surgical ligation of vessels, use of mono- or bipolar electric scalpels, as well as advanced electrical energy devices or ultrasonic energy for sealing of vessels. The technique used depends on the surgeon’s experience and the local availability of the technology. Late complications result from injury or removal of adjacent structures, such as the parathyroid glands and the recurrent laryngeal nerve, resulting in hypoparathyroidism or dysphonia, respectively. These complications may result from direct manipulation of the structures, as well as hyperthermia associated with the use of hemostasis equipment.^4^ The use of advanced or ultrasonic bipolar energy as a hemostatic method in thyroid surgery can lead to a decrease in operative time, without an impact on the occurrence of complications.^5^, ^6^, ^7^, ^8^, ^9^ However, studies have compared bipolar or ultrasonic advanced energy versus conventional energy with each other but have not included the first two in the same analysis or controlled for eventual differences between centers. The aim of our study is to compare the performance of three technologies (conventional electrical, advanced and ultrasonic) at the same institution and with the same surgical teams.

## Methods

Patients submitted to surgery between January 1, 2015 and December 31, 2016 were analyzed. During this period, all patients undergoing surgical procedures were prospectively included in an institutional database but were not randomized regarding the type of intervention. It is, therefore, a prospective cohort study. The following inclusion criteria were considered for the present study: total thyroidectomy using an electric scalpel or other types of energy and absence of previous surgery, radiotherapy or radioiodine therapy. The exclusion criteria were: partial thyroidectomy or thyroidectomy totalization; concomitant procedure, including recurrent compartment emptying and parathyroidectomy; use of multiple energy sources in the same patient; use of sternotomy and use of video-assisted or robotic access. Serum parathyroid hormone (PTH) levels and laryngeal mobility assessment by nasofibrolaryngoscopy (NFL) were performed in the preoperative evaluation. All procedures were performed under general anesthesia using a wired cannula. The hemostasis technique was the personal choice of the surgeon responsible for the procedure. In the postoperative period, PTH was collected right after surgery and seven days after the procedure and NFL assessment was performed in the patients 10 days after surgery. Postoperative followup encompassed at least 6 months, with PTH measurement at 6 months and a new assessment of vocal fold mobility at 3 and 6 months, if there was any alteration found during the first examination.

Surgery time was defined as the time between the skin incision and the complete incision closure. The occurrence of vocal fold paralysis was recorded through postoperative laryngoscopy, and hypoparathyroidism was considered if the postoperative PTH level was <12 pg/milliliter (pg/mL). In patients with a PTH level <12 pg/mL 6 months after the procedure, hypoparathyroidism was considered definitive.

The statistical analysis was performed using the Stata 14.2 statistical package (Stata Corp., College Station - TX, USA). Continuous variables are described by mean and standard deviation (SD), while categorical variables are described by their frequency. Multiple imputation was performed for missing data. Means were compared by Student's *t* test. Multiple simultaneous comparisons of means between groups were performed by analysis of variance (ANOVA). The association between continuous variables was established by linear regression when the variable of interest was continuous or by logistic regression when the variable of interest was categorical. These models are demonstrated by the variable of interest coefficient value and 95% Confidence Interval (CI). A value of *p* ≤ 0.05 was considered statistically significant. This study was carried out after approval by the Research Ethics Committee (Opinion number 2,904,573).

## Result

**Figure 1:**
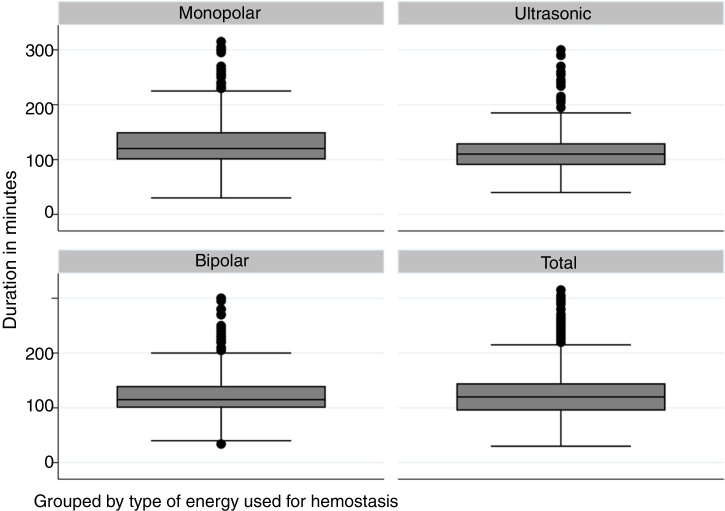
Distribution of operative time in the cohort analyzed according to the type of energy used and the total number of patients.

A total of 834 patients were submitted to total thyroidectomy during this period and met the inclusion criteria in this study. There were 661 women (79.3%) and 173 men (20.7%). The mean age was 46 years with a standard deviation of 13.5 years, ranging from 14 to 87 years. The Body Mass Index (BMI) ranged from 14.0 to 46.9 (mean of 27.1 and SD of 4.9). The diagnosis was malignant neoplasia in 528 patients (63.3%) and benign disease (goiter or benign tumor) in 306 patients (36.7%). Regarding the type of energy, the monopolar electric scalpel was used in 280 patients (33.6%), advanced bipolar energy in 210 patients (25.2%) and ultrasonic energy in 344 patients (41.3%). The demographic characteristics of patients according to the type of energy used are shown in Table 1. Fig. 1 shows the distribution of operative time according to the type of energy.

**Table 1:** Demographic characteristics of the assessed patients.

Variable	Values	Type of energy		
		Electrical monopolar	Electrical bipolar	Ultrasonic
Gender	Female	231	161	269
	Male	49	49	75
Age (years)	Mean (SD)	47.62 (13.62)	45.84 (13.81)	44.69 (13.11)
BMI	Mean (SD)	27.07 (4.78)	27.33 (4.96)	26.97 (4.94)
Malignant	No	114	76	116
	Yes	166	134	228
Transient hypocalcemia	No	194	168	271
	Yes	86	42	73
Vocal fold paralysis	No	272	203	319
	Yes	8	7	25

An initial comparison between duration of the procedure by type of energy used was significantly shorter with bipolar or ultrasonic energy use (Table 2). All hemostasis methods were used by each of the ten surgeons, but there was no difference in duration of the procedure according to the surgeon when compared by ANOVA. For patients submitted to electric scalpel use, the mean time was 124.84 min (SD  = 43.22 min; *p* = 0.298); for advanced bipolar energy use, the mean time was 112.09 min (SD = 27.73 min; *p* = 0.113) and for ultrasonic energy use, the mean time was 116.52 min (SD = 32.32 min; *p* = 0.240). In a linear regression model, gender, malignancy diagnosis, and type of energy were significant for procedure duration (Table 3).

**Table 2:** Comparison of the mean duration of surgeries measured in minutes according to the type of energy used.

Type of energy	Monopolar energy	Bipolar energy
Bipolar energy	−12.75 (*p* = 0.002)	
Ultrasonic energy	−8.32 (*p* = 0.035)	4.43 (*p* = 0.447)

**Table 3:** Linear regression model for operative time in patients submitted to total thyroidectomy.

Variable	Value	Coefficient	95%CI	*p* value
Gender	Male	1		
	Female	−18.31	−24.99 to −11.62	<0.001
Age		−0.07	−0.277 to 0.129	0.477
BMI		0.441	−0.110 to 0.993	0.117
Malignant	No	1		
	Yes	−5.99	−11.61 to −0.390	0.036
Type of energy	Monopolar	1		
	Bipolar	−13.73	−20.786 to −6.663	<0.001
	Ultrasonic	−8.88	−15.134 to 2.628	0.005

Length of hospital stay ranged from 1 to 12 days. There was no significant difference in length of stay according to the type of energy used (Table 4). There were 201 cases of acute hypocalcemia (24.1%) and 16 cases (1.9%) of definitive hypocalcemia. Transient vocal fold paresis or paralysis occurred in 40 patients (4.8%). When considering acute postoperative complications as an outcome of interest, the occurrence of acute hypocalcemia is influenced by the type of energy used (Table 5). No variables significantly associated with the risk of vocal fold motility changes or significant late sequelae were identified.

**Table 4:** Comparison of length of hospital stay according to the type of energy.

Type of energy	Monopolar energy	Bipolar energy
Bipolar energy	−0.132 (*p* = 0.178)	
Ultrasonic energy	−0.067 (*p* = 0.606)	0.069 (*p* = 0.595)

**Table 5:** Logistic regression model for acute hypocalcemia outcome in patients submitted to total thyroidectomy.

Variable	Value	Coefficient	95%CI	*p* value
Gender	Male	1		
	Female	0.60	0.155 to 1.051	0.008
Age		<0.001	−0.012 to 0.012	0.923
BMI		0.025	−0.008 to 0.057	0.137
Malignant	No	1		
	Yes	−0.009	−0.346 to 0.327	0.957
Type of energy	Monopolar	1		
	Bipolar	−0.545	−0.972 to −0.118	0.012
	Ultrasonic	−0.483	−0.851 to −0.115	0.010

## Discussion

Technological innovations are constantly emerging and their incorporation into surgical practices may or may not result in significant benefits for the patient.^10^, ^11^ Therefore, the results of these changes must be critically and constantly evaluated.

In the thyroid surgery area, the two most recently introduced innovations with a major impact were the use of intraoperative recurrent laryngeal nerve monitoring and new types of energy for hemostatic control. This is especially important because of the risk of postoperative bleeding, which is up to 2%.^4^

In a meta-analysis assessing 14 prospective randomized trials with 2293 patients comparing ultrasonic forceps with monopolar or advanced bipolar energy, a significant benefit was observed regarding operative time reduction, with a difference of 22.43 min favoring the former.^12^ In all evaluated studies, there was an effect of the forceps on operative time reduction, with differences between time averages ranging from 10 to 61 min.^13^, ^14^ This same effect was observed in our series; however, without the magnitude reported by the meta-analysis, albeit with statistical significance. We also failed to observe the reduction in hospital stay seen in this meta-analysis. In our patients, the energy modality did not affect the length of hospital stay, which is on average quite short. Regarding postoperative complications, the use of advanced harmonic or bipolar scalpel was associated with a significant reduction in the risk of hypocalcemia and operative time in a meta-analysis of 35 prospective randomized studies involving 2865 patients, but with an increased incidence of recurrent laryngeal nerve paresis/paralysis when compared with the conventional technique.^15^ The decreased incidence of hypocalcemia was also observed in our series, but we did not observe any difference regarding recurrent laryngeal nerve paresis/paralysis in our patients.

The comparison between advanced bipolar and conventional electrical energy shows advantages for the former. In a series of 517 consecutive patients undergoing total thyroidectomy for benign disease, there was a statistically significant reduction in postoperative drainage volume, length of hospital stay, transient hypoparathyroidism, recurrent laryngeal nerve paresis/paralysis, and cervical hematoma.^16^ These results are comparable to ours regarding operative time and hypoparathyroidism. In our country, there was a randomized multicenter study that compared the use of ultrasonic scalpel with the conventional technique in 216 patients, which demonstrated a benefit of reduced operative time with the first modality.^9^ In this case, postoperative complications were not analyzed.

In a meta-analysis of 7 studies analyzing 1473 patients, comparing the use of ultrasonic with advanced bipolar energy, a statistically significant advantage of the former in relation to operative time was observed, with comparable results regarding blood loss, length of hospital stay and postoperative serum calcium level.^17^

In a meta-analysis involving both prospective randomized studies and prospective cohorts, a significant decrease in operative time was observed when using bipolar or ultrasonic energy compared to the conventional energy.^18^ In this analysis, we also compared the operative time of the ultrasonic scalpel with bipolar energy, with significant advantage of the former.

In this series, the outcome of recurrent laryngeal nerve paralysis showed no significant difference between the groups. It is noteworthy that this study included patients submitted to partial thyroidectomy and a significant reduction in operative time was also observed in these patients.^19^

## Conclusion

In our series, the use of bipolar or ultrasonic energy allowed a significant reduction in operative time, in agreement with previous reports and meta-analyses. However, such reduction was close to the lower limit of those reported in the literature. The occurrence of acute postoperative hypocalcemia also showed a significant reduction with the use of ultrasonic or bipolar energy, reducing the need for calcium replacement, but permanent hypocalcemia was not affected by the technique used. Finally, in our series, the technique used showed no impact on the length of hospital stay or changes in the ultimate recurrent laryngeal nerve function. Therefore, the benefits of the ultrasonic or bipolar scalpel in our series were smaller than those shown in the meta-analyses of international studies.

## Conflicts of interest

The authors declare no conflicts of interest.
